# Trench Mouth

**Published:** 1919-06

**Authors:** Guy A. Karr


### "TRENCH MOUTH"

BY GUY A. KARR, D.C., N.G.

"Trench Mouth," as it is commonly called on the West front, is an accute ulcerative disease very prevalent among soldiers engaging in trench warfare and very prevalent after the soldiers return from a tour of trench duty.

It is closely related to the disease known as Vincent's Angina, and can best be described by discussing this disease. Vincent's Angina and the disease known as acute ulcerative Gingivitis, or interstitial Gingivitis, are believed by many writers to be very closely related and is described by Vaughan, Gilmer, Black and others, while Vincent's Angina, in the majority of cases, occurs as a disease of the tonsils and fauces, but many include the gum margins and associated tissues.

Mr. W. Eichmeyer states that the Bacillus Fusiformis, a spindle-shaped spirochete, is always present, but at times others organisms predominate. He states that not only Vincent's Angina and Ulcerative Gingivitis are identical in their Etiology, but that Noma is due to the same organisms.

The affection is characterized by the formations of ulcers. The onset is sudden. The earliest symptoms are indicated by a slight malaise, which is soon followed by rapid ulceration, at first confined to the gingivae, usually about two or three of the anterior teeth on both jaws, simultaneously and in corresponding localities later it is extended to the gums of other teeth. The lingual margins and festoons do not at first participate in the inflammatory process, but later the festoons are destroyed and deep pockets are formed in the interproximal spaces. In twenty-four hours after the patient's attention is called to his condition of his gums, the parts present the appearance of having been gnawed away until most of the gum tissue overlying the aveolar process immediately surrounding the teeth has been destroyed, the part attacked has soft, thick margins.

The eroded parts form pockets which are filled in with a grayish pasty mass, similar to syphilitic ulcers of the mouth. When this mass is removed a granulation surface is exposed which bleeds easily and is very painful to the touch.

The mucous membrane for a short distance from the ulcerative margins is of a dark red hue, as a result of the congestion. In many cases the gums are destroyed sufficiently to uncover the teeth down to the aveolar border. The breath of the patient is fetid, the saliva ropy and greatly in excess. The temperature in the early stages ranges about 101 degrees; the patient is nervous and constipated.

Without treatment the ulceration does not go deeper than the aveolar process, but when the case is neglected the entire mucous of the mouth in a degree is affected—that is, to the extent of becoming red and tender. The ulceration seems to be limited to the areas indicated, and after the destruction of the mature tissue has ceased the granulations only appear to be destroyed, leaving the pasty mass covering. It is well at this point to dwell somewhat on the Etiology of this disease. Brophy says: This condition is found frequently in poorly nourished and unclean individuals. Lack of attention to the teeth is the chief Etiological factor. It is evidently due to disturbed nutrition and oral uncleanliness, and from this it is easy to see how it could be very prevalent in the trenches, as the troops in many cases are in the trenches for several days with poor food and poor facilities for the proper care of the teeth.

A number of different drugs may be used in the treatment; the first thing is to prescribe a saline cathartic, using an alkaline mouth wash. Black suggests washing the ulcers with a strong solution of sodium chloride. His method is to take the bulb syringe and put all the pressure possible in order to have a strong spray directed against the ulcer, thereby washing away all the gray mass. He also advises the use of peroxide of hydrogen as a mouth wash, finally swabbing the ulcers with a solution made up of equal parts of iodine and aconite. With this treatment the condition should be cleared in from four days to two weeks.

Major Pearson suggests the application of ferric chloride. Four cases of this disease have been found in the 129th Infantry, and all indications are that it is contagious, as the four cases have come from two companies. All authorities agree that it is contagious, care should be used in thoroughly sterilizing instruments.—*Journal of Military Dental Surgeons.*

